# LINC02774 inhibits glycolysis in glioma to destabilize HIF‐1α dependent on transcription factor RP58

**DOI:** 10.1002/mco2.364

**Published:** 2023-09-11

**Authors:** Yuanbing Chen, Yating Liu, Jianbing Xiong, Lianlian Ouyang, Miao Tang, Chao Mao, Liling Li, Desheng Xiao, Shuang Liu, Zhen Yang, Jun Huang, Yongguang Tao

**Affiliations:** ^1^ Department of Neurosurgery Third Xiangya Hospital, Central South University Changsha Hunan China; ^2^ Department of Neurosurgery Xiangya Hospital, Central South University Changsha China; ^3^ National Clinical Research Center for Geriatric Disorders Xiangya Hospital, Central South University Changsha Hunan China; ^4^ Key Laboratory of Carcinogenesis and Cancer Invasion Ministry of Education, Central South University Hunan China; ^5^ NHC Key Laboratory of Carcinogenesis (Central South University), Cancer Research Institute Central South University Changsha Hunan China; ^6^ Department of Emergency Xiangya Hospital, Central South University Changsha Hunan China; ^7^ Department of Pathology Xiangya Hospital, Central South University Changsha Hunan China; ^8^ Department of Oncology Xiangya Hospital, Central South University Changsha China; ^9^ Shanghai Key Laboratory of Medical Epigenetics Fudan University Shanghai China

**Keywords:** glioma, glycolysis, LINC02774, RIEMR, RP58

## Abstract

Glioma, the most common of malignant tumors in the brain, is responsible for the majority of deaths from primary brain tumors. The regulation of long noncoding RNAs (lncRNAs) in HIF‐1α‐driven tumor development remains unclear. LINC02774 is a nuclear lncRNA and that it is being reported for the first time in this study. We found the downregulation of LINC02774 in glioma and decreased with the degree of malignant, with its expression showing a negative correlation with the relative index of enhanced magnetic resonance (RIEMR). RIEMR‐associated LINC02774 was found to inhibit glycolysis by modulating the hypoxia pathway rather than the hypoxia response itself. LINC02774 interacted with its neighboring gene, RP58 (ZBTB18), to enhance the expression of PHD3, which catalyzed HIF‐1α hydroxylase and ubiquitination, leading to the downregulation of HIF‐1α expression. We also found that the function of LINC02774, dependent on PHD3, was diminished upon RP58 depletion. Notably, higher expression of RIEMR‐associated LINC02774 was associated with a favorable prognosis. In conclusion, these findings reveal the role of RIEMR‐associated LINC02774, which relies on its neighbor gene, RP58, to regulate the hypoxia pathway as a novel tumor suppressor, suggesting its potential to be a prognostic marker and a molecular target for the therapy of glioma.

## Graphical abstract

RIEMR‐associated LINC02774 interacts with RP58, leading to the upregulation of PHD3 expression, which, in turn, facilitates the hydroxylation and ubiquitination of HIF‐1α. Consequently, the degradation of HIF‐1α is promoted, resulting in the downregulation of GLUT1 and VEGF expression. Ultimately, this leads to the inhibition of the glycolysis pathway and the progression of glioma

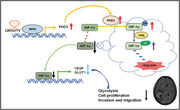

## 1 INTRODUCTION

Glioma is the most prevalent kind of malignant brain tumor.^1^ According to the WHO grading system, glioma is categorized into four classes, which aids in assessing its degree of malignancy. However, treating various types of glioma remains challenging.^2^, ^3^, ^4^ Thus, it is critical to further explore the molecular mechanisms underlying glioma to enhance therapeutic strategies and identify novel prognostic molecular markers.

The human genome encodes numerous lncRNAs that have been identified to modulate the progress of tumors.^5^, ^6^, ^7^ In gliomas specifically, lncRNAs have been found to have significant implications in tumor initiation, progression, and therapeutic resistance regulation.^8^ Magnetic resonance imaging (MRI) enables the assessment of enhancement parameters in postenhanced T1‐weighted images, providing information on tissue vascularity and perfusion, as well as the influence of pathological angiogenesis.^9^ Given that a higher degree of malignancy is associated with a more prominent signal enhancement in postenhanced T1‐weighted images, we calculated the relative index of enhanced magnetic resonance (RIEMR). However, the potential connection between lncRNAs and RIEMR in glioma remains unknown.

Hypoxia, a prevalent characteristic in solid tumors, is closely linked to malignant progression across various cancer.^9^, ^10^ Hypoxia‐inducible factors (HIFs), which consist of three family members, associate with the procession of the adaptive response to hypoxia.^11^ The target genes regulated by HIF‐1 are well established to be involved in various aspects of tumorigenesis.^12^ The Warburg effect, a common phenomenon in cancer, refers to altered glycolysis, and HIF‐1 is responsible for determining whether glucose is consumed through glycolysis or oxidation.^13^, ^14^, ^15^ HIF‐1 is made up of α‐ and β‐subunits, and its functional activity depends on the stability and nuclear translocation of the HIF‐1 subunit.^16^, ^17^ It is commonly observed that solid tumors exhibit elevated levels of HIF‐1α, which is also related to the clinical outcome in various types of cancer.^18^, ^19^ Additionally, proline hydroxylases (PHDs) play crucial roles in regulating the process of HIF‐1α hydroxylation, resulting in HIF‐1α ubiquitination and subsequent degradation.^20^ Although a previous study identified dysregulated lncRNA expression or lncRNA interactions influenced by HIF‐1α under hypoxia,^16^ the definite impact of lncRNAs on HIF‐1α is not completely understood.

In the development of the brain and the differentiation of neurons, the transcriptional repressor Zinc Finger and BTB Domain Containing 18 (ZBTB18; formerly RP58) is essential, such as the deletion of RP58 leads to microcephaly, cerebellum hypoplasia, and corpus callosum agenesis in the mouse.^21^, ^22^ And the RP58 also acts as a crucial role in tumor.^23^, ^24^ Notably, the RP58 has been found that downregulated in glioma, which indicated that RP58 function as a tumor suppressor in the glioma.^25^ However, it is still unknown how RP58 affects gliomas.

Here, we found the LINC02774 is an lncRNA and that it is being reported for the first time in this study. And the role of LINC02774 in regulating the stability of HIF‐1α and its impact on glioma progression was explored. Our investigation revealed that LINC02774 expression is downregulated in glioma, and its levels are inversely correlated with RIEMR. Furthermore, we demonstrated the significance of the RIEMR‐associated LINC02774 in modulating the stability of HIF‐1α, with this process relying on the transcription factor RP58 and the HIF‐1α regulator PHD3.

## 2 RESULTS

### 2.1 RIEMR‐associated LINC02774 is downregulated in glioma

**FIGURE 1:**
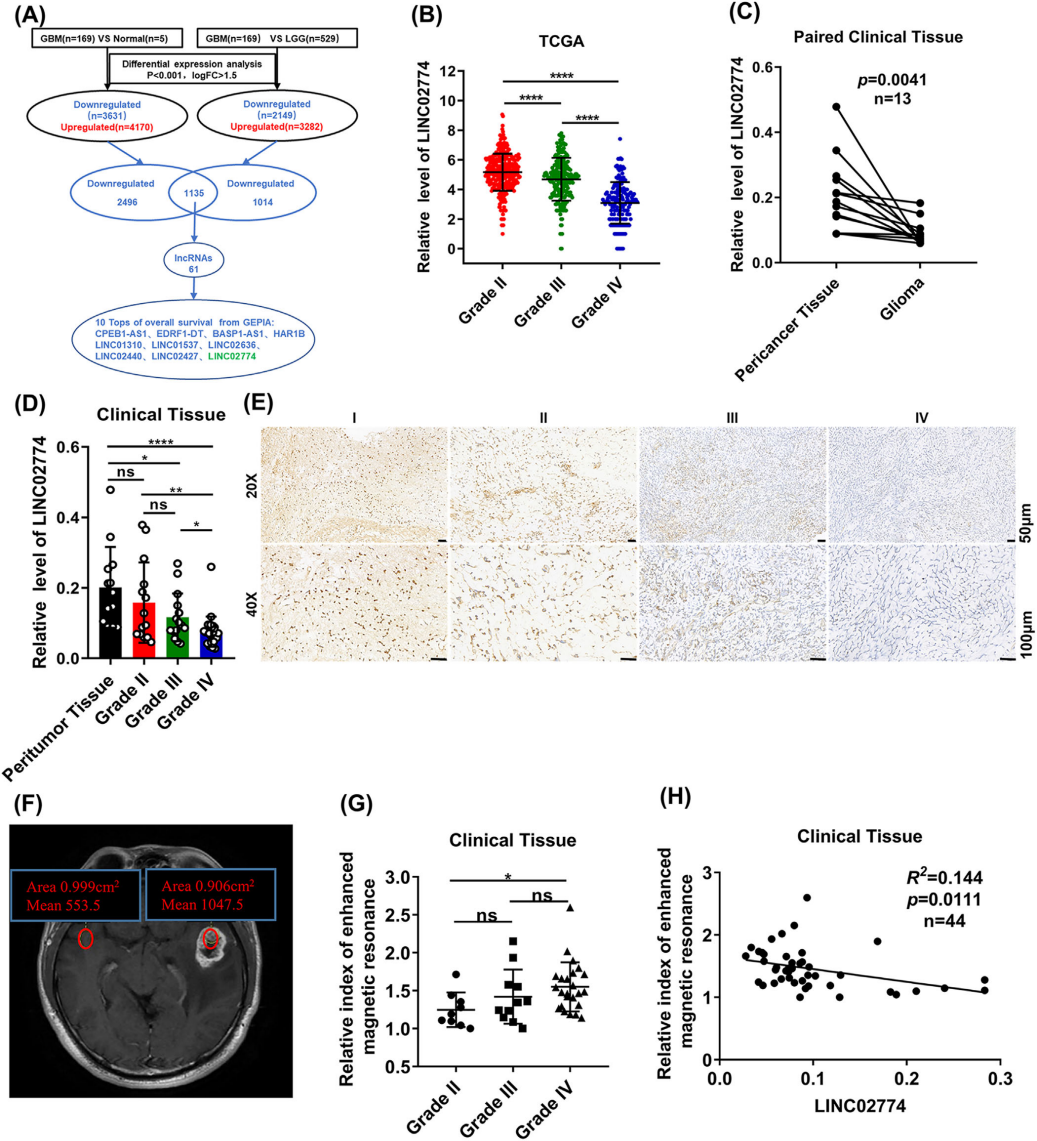
RIEMR‐associated LINC02774 is downregulated in glioma. (A) Schematic overview to explore “Glioma‐associated lncRNAs” in glioma. We performed a screening strategy, and 61 filtered out downregulated lncRNAs from the TCGA datasets. Then, combining the Kaplan–Meier analysis from the GEPIA, the 10 top significant lncRNAs were selected. (B) The expression of LINC02774 in different WHO grades (II, *n* = 311; III, *n* = 208; IV, *n* = 166). (C) qRT‐PCR demonstrated decreased LINC02774 expression in 13 pairs of glioma compared to pericancer tissues. (D) qRT‐PCR detected the LINC02774 expressed in clinical tissues (pericancer tissues, *n* = 13; II, *n* = 14; III, *n* = 15; IV, *n* = 27). (E) ISH illustrated that LINC02774 is located in the nucleus and revealed the decrease of LINC02774 with the degree of malignant. (F–H) The RIEMR measured from magnetic resonance imaging (MRI) (F) in glioma of different WHO grades (II, III, and IV) (G). Correlations between RIEMR and LINC02774 expression in glioma tissues (H). GBM: glioblastoma, LGG: low‐grade glioma, qRT‐PCR: quantitative real‐time PCR, RIEMR: relative index of enhanced magnetic resonance, RIEMR: relative index of enhanced magnetic resonance.

To investigate glioma‐associated lncRNAs, we implemented a screening strategy utilizing the The Cancer Genome Atlas (TCGA) gene expression profile data. First, we examined the genes that were differentially expressed between normal brain tissue and glioblastoma (GBM). Additionally, we identified the differentially expressed genes between low‐grade glioma (LGG) and GBM to identify genes linked to glioma occurrence and progression. Subsequently, the downregulated lncRNAs were selected and performed survival analysis using GEPIA. From this analysis, we selected the top 10 lncRNAs with significant implications (Figures 1A and S1A). Long intergenic nonprotein coding RNA2774 (LINC02774), is a lncRNA on human chromosome 1q44 and its function has not yet been explored in cancer. Although LINC02774 was not the most significant among the downregulated lncRNAs, it demonstrated highly specific expression in brain tissue and exhibited lowly expression levels in glioma, correlating with the malignancy degree compared to other addressed lncRNAs (Figures 1B and S1B‐D).

In addition, we observed downregulation of LINC02774 in 13 glioma specimens compared to matched peritumor tissues (Figure 1C). And in a larger cohort of 56 glioma tissues, the expression of LINC02774 decreased with the increasing WHO grade (Figure 1D). Additionally, we performed in situ hybridization (ISH) on paraffin sections of glioma and observed the highest expression of LINC02774 in grade I glioma tissues, whereas the lowest expression was observed in grade IV glioma tissues. Importantly, LINC02774 was primarily localized in the nucleus rather than the cytoplasm (Figure 1E). The expression of LINC02774 was detected in glioma cell lines (U251, U87‐MG, HS683), we found that HS683, derived from a Grade II patient, exhibited the highest expression of LINC02774 (Figure S1E). Similar to clinical tissue samples, LINC02774 was predominantly localized in the nucleus in glioma cell lines (Figure S1F). Further, abnormal DNA methylation is related to the tumor suppressor genes are silenced in some cancer cells.^26^, ^27^ The TCGA gene expression profile data and methylation levels were analyzed and discovered the expression of LINC02774 was negative correlated to the methylation level of CpG sites (Figure S1G).

To investigate the relationship between LINC02774 and the degree of malignancy on MRI, we calculated the RIEMR based on the characteristic that higher malignancy corresponds to a more prominent signal image on postenhanced T1‐weighted MRI (Figure 1F). We confirmed that RIEMR values were associated with glioma grades (Figure 1G). Interestingly, we found the expression of LINC02774 was negative correlated to the RIEMR values (Figure 1H). The ROC curve analysis further supported the relationship between LINC02774 expression and RIEMR values, indicating that the higher expression of LINC02774 was related to the weaker signal images in the postenhanced T1‐weighted MRI (Figure S1H). Additionally, univariate analysis revealed significant associations between RIEMR values, WHO grade, and LINC02774 expression (*p* < 0.05, Table 1).

**TABLE 1:** Correlation between LINC02774 expression and clinicopathological parameters in glioma tissues (*χ* ^2^ test).

		LINC02774			
	*n*	Low	High	*χ* ^2^	*p*
Age				0.6437	0.4224
<50	27	12	15		
≥50	29	16	13		
Gender				0.6857	0.4076
Male	35	16	19		
Female	21	12	9		
WHO Classification				6.171	0.0457
II	14	4	10		
III	15	6	9		
IV	27	18	9		
Location				0.8205	0.6635
Left	24	12	12		
Right	26	14	12		
Middle	6	2	4		
RIEMR				9.091	0.0026
Low	22	6	16		
High	22	16	6		

RIEMR: relative index of enhanced magnetic resonance.

These findings demonstrate that LINC02774 is downregulated in glioma and associated with the RIEMR, suggesting its potential role as a tumor suppressor in glioma.

### 2.2 Overexpression of RIEMR‐associated LINC02774 inhibits the tumorigenesis of glioma

**FIGURE 2:**
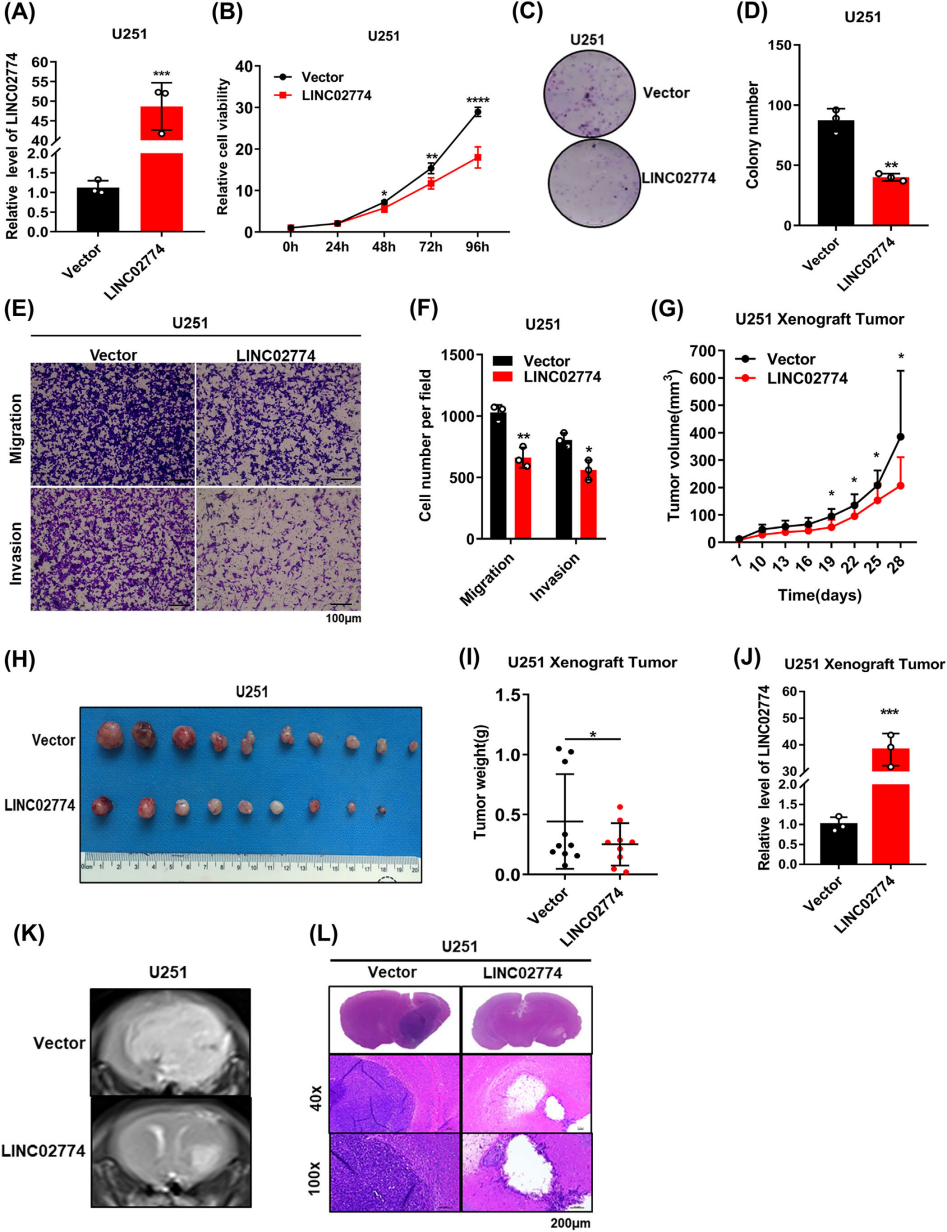
Overexpression of RIEMR‐associated LINC02774 inhibits the tumorigenesis of glioma. (A) Identity the expression of LINC02774 in U251 cells overexpressing LINC02774. (B) Cell viability in U251 cells overexpressing LINC02774 evacuated by MTS. (C, D) Colony formation assay was performed in U251 cells stably overexpressing LINC02774 (C), colony number counted by ImageJ (D). (E, F) LINC02774 overexpression inhibited the migration and invasion of U251 cells (E). ImageJ was performed to count cells to evaluate invasion and migration ability (F). (G–I) Xenograft tumors. Tumor volume (G), images (H), and weights (I) were recorded (*n* = 10 for each group). (J) The expression of LINC02774 in xenograft tumors. (K, L) Orthotopic xenograft models were established. Tumor formation was monitored using MRI (K) and H&E (L). H&E: hematoxylin and eosin.

To investigate the function of RIEMR‐associated LINC02774, the expression of LINC02774 was examined in different glioma cell lines. We found higher expression of LINC02774 in HS683 and U87‐MG compared to U251 (Figure S1E). To further elucidate the function of LINC02774, we overexpressed LINC02774 in the U251 glioma cell line using lentiviral vectors. Successful overexpression of LINC02774 was confirmed (Figure 2A). Overexpression of LINC02774 resulted in a weakened proliferative ability of U251 cells (Figure 2B) and a significant reduction in colony formation (Figure 2C and D). Glioma is known for its aggressive growth and invasive behavior, partly attributed to EMT induction in cancer cells.^28^ We further demonstrated that overexpression of LINC02774 decreased the protein levels of mesenchymal markers (Figure S2A). Consistently, overexpression of LINC02774 significantly impaired the invasion and migration capabilities of U251 cells (Figure 2E and F).

The xenograft models was used to investigate the function of LINC02774 in vivo. Injection of U251‐LINC02774 cells resulted in significantly reduced tumor sizes, volumes, and weights compared to U251‐Vector cells after 28 days of growth (Figures 2G‐J and S2B). Furthermore, to evaluate LINC02774's function in relation to glioma within the brain tissue, we conducted orthotopic xenograft models and measured tumor sizes using MRI and histological analysis (H&E). Consistently, LINC02774 overexpression led to significant suppression of glioma sizes compared to U251‐Vector cells in the cerebral region (Figures 2K and L and S2C). Taken together, our findings demonstrate that LINC02774 inhibits cell proliferation, colonization, invasion, migration, and tumor growth, indicating that the RIEMR‐associated LINC02774 functions as a tumor suppressor in glioma.

### 2.3 Knockdown of RIEMR‐associated LINC02774 promotes the tumorigenesis of glioma

**FIGURE 3:**
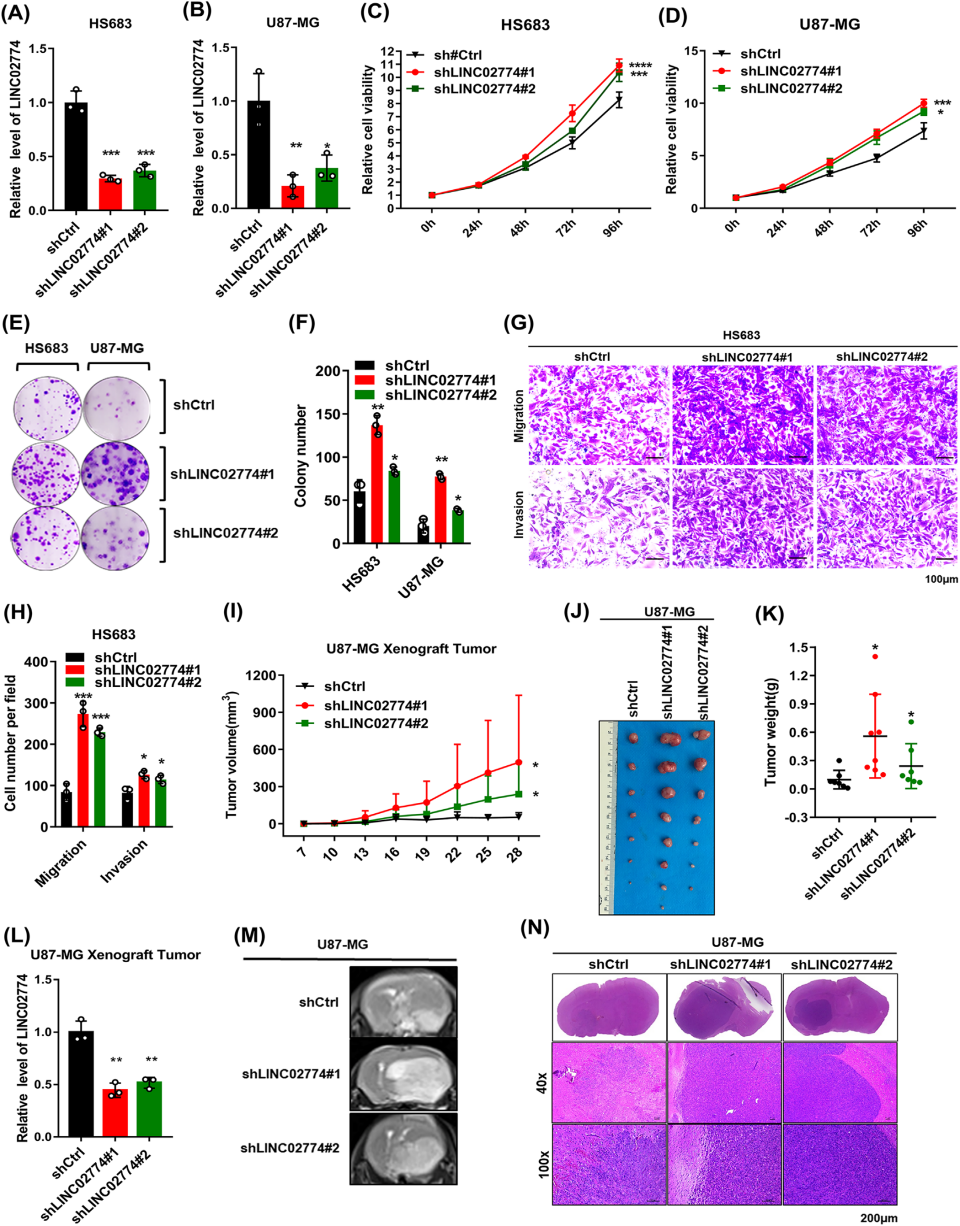
Knockdown of RIEMR‐associated LINC02774 promotes the tumorigenesis of glioma. (A, B) The level of LINC002774 in HS683 (A) and U87‐MG (B) were detected. (C, D) The cell viability in HS683 (C) and U87‐MG (D) cells were detected. (E, F) The colony formation of HS683 and U87‐MG (E) cells after the knockdown of LINC02774 was measured. LINC02774 promoted colony formation in glioma cells, counted by ImageJ (F). (G, H) LINC02774 knockdown improved the migration and invasion of HS683 cells. (I–K) Tumor volume (I), tumor formation (J), and tumor weight (K) in nude mice. (L) Expression of LINC02774 measured by qRT‐PCR in xenograft tumors. (M, N) The xenograft tumors in the cerebral region. U87‐MG cells with knockdown of LINC02774 and control cells were injected, and tumor formation was monitored using MRI (M) and H&E (N).

The stable knockdown of LINC02774 using lentivirus in U87‐MG and HS683 were constructed. The efficiency of the sequences for knockdown was identified (Figure 3A and B). The knockdown of LINC02774 in U87‐MG and HS683 cells significantly promoted cell growth (Figure 3C and D). Additionally, the knockdown of LINC02774 significantly enhanced colony formation in both U87‐MG and HS683 cells (Figure 3E and F) and also significantly promoted invasion and migration in the HS683 cell line (Figure 3G and H).

To further validate the function of LINC02774 in vivo, U87‐MG cells were injected into subcutaneously. We discovered that the depletion of LINC02774 expression significantly promoted the growth of tumor (Figures 3I–L and S3A). Furthermore, orthotopic xenograft model was performed to demonstrate that the knockdown of LINC02774 in U87‐MG cells promoted tumor formation in brain (Figures 3M and N and S3B). Collectively, these findings reveal that LINC02774 could inhibit glioma progression in vitro and in vivo.

### 2.4 RIEMR‐associated LINC02774 represses the pathways of hypoxia and glycolysis

**FIGURE 4:**
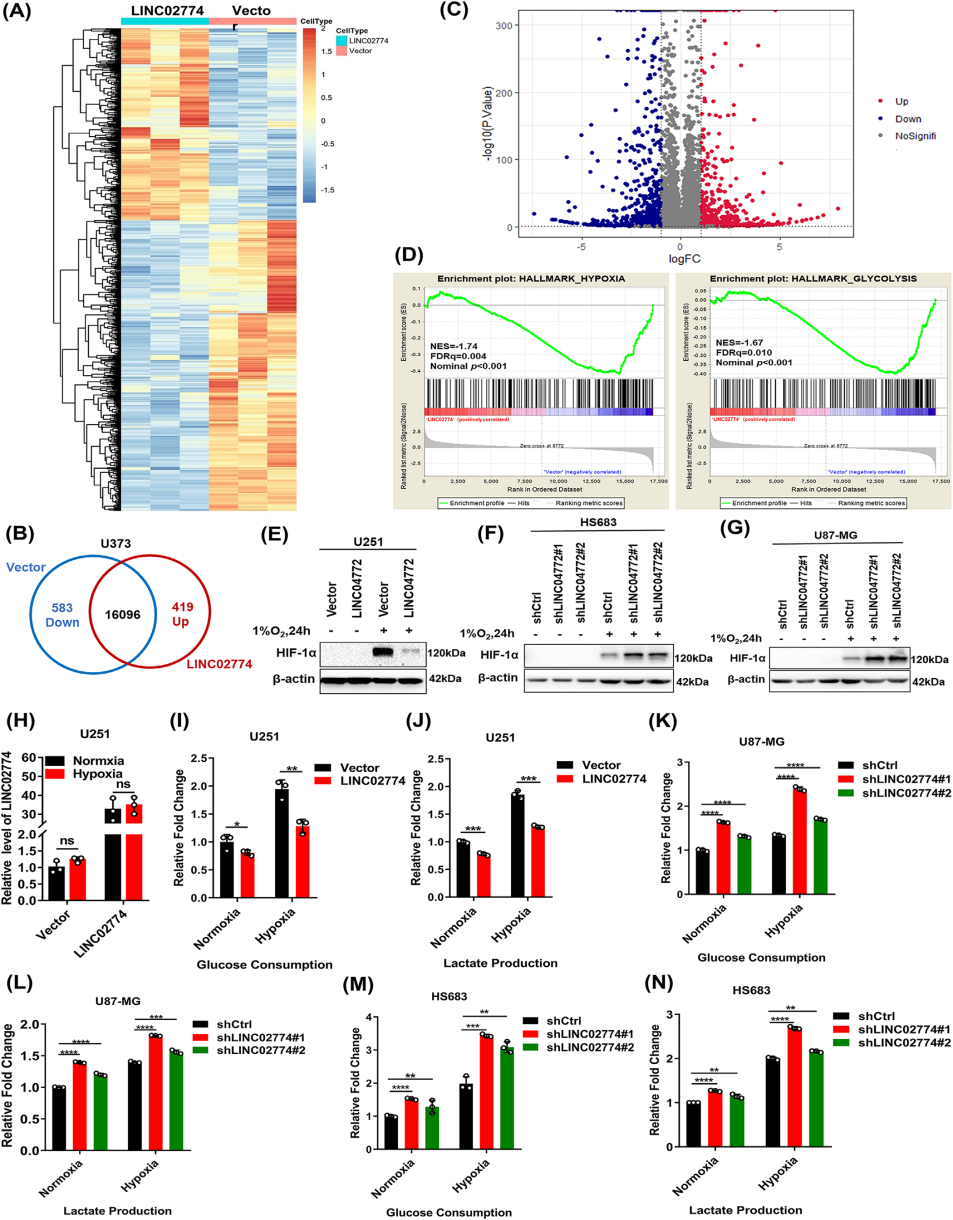
RIEMR‐associated LINC02774 represses the pathways of hypoxia and glycolysis. (A) The differential expressed genes induced by stably overexpressing LINC02774 in U251 cells, with red indicating a high expression level of mRNA. (B) LINC02774 induced 583 genes downregulated and 419 genes upregulated in U251 cells. (C) Volcano plot indicating significantly differentially expressed genes. (D) GSEA results revealed that LINC02774 highly downregulated the hypoxia and glycolysis‐related gene sets. (E) The effect of stably overexpressing LINC02774 on the HIF‐1α protein level in U251 cells exposed to normoxia or hypoxia for 24 h. (F, G) LINC02774‐knockdown increased the protein level of HIF‐1α in HS683(F) and U87‐MG(G). (H) The expression of LINC02774 did not alter in normoxia and hypoxia. (I, J) The level of glucose (i) and lactate (j) detected in culture medium of U251 cells overexpressing LINC02774. (K–N) U87‐MG (K, L) and HS683(M, N) cells with LINC02774 depletion cultured in 21% or 1% O_2_ for 24 h, and measured the glucose (K, M) and lactate (L, N) levels in the culture medium.

To investigate the potential mechanisms of LINC02774, we performed RNA‐seq in U251 cells overexpressing LINC02774 compared to the control vector. Following data processing, we identified 1002 genes that exhibited differential expression between the two groups (Figure 4A and B). A volcano plot visualized these changes, revealing 583 downregulated genes and 419 upregulated genes (Figure 4C). In order to learn more about the biological processes and pathways connected to these differentially expressed genes, we carried out a functional classification and enrichment analysis using the gene ontology database. The results revealed significant enrichment in pathways related to cellular processes, cellular components, and biological regulation (Figure S4A and B). To evaluate the enrichment of differentially expressed genes in various biological processes, KEGG pathway analysis was also carried out (Figure S4C and D). These analyses provided evidence that LINC02774 is closely associated with human diseases.

We used qRT‐PCR to confirm the differentially expressed genes, and the results were in agreement with the RNA‐seq data (Figure S4E and F). LINC02774 expression has a negative connection with the hypoxia and glycolysis pathways, according to gene set enrichment analysis (GSEA) (Figure 4D). In subsequent validation experiments, overexpression of LINC02774 in U251 glioma cells significantly decreased the protein levels of HIF‐1α (Figure 4E). Conversely, depletion of LINC02774 in U87‐MG and HS683 cells significantly enhanced the protein level of HIF‐1α (Figure 4F and G). Interestingly, the expression of LINC02774 was not significantly different between hypoxic and normoxic conditions (Figure 4H), indicating that its expression might not be influenced by oxygen concentration, unlike previously reported hypoxia‐inducible lncRNAs.^16^, ^29^ Furthermore, we found that overexpression of LINC02774 in U251 glioma cell lines led to decrease glucose consumption and lactate production, both under hypoxic and normoxic conditions (Figure 4I and J). Conversely, the depletion of LINC02774 resulted in increasing glucose consumption and lactate production (Figure 4K–N). These results indicate that LINC02774 acts as a tumor suppressor by regulating the hypoxia and glycolysis signaling pathway in glioma.

### 2.5 RIEMR‐associated LINC02774 promotes the degradation of HIF‐1α by upregulating PHD3

**FIGURE 5:**
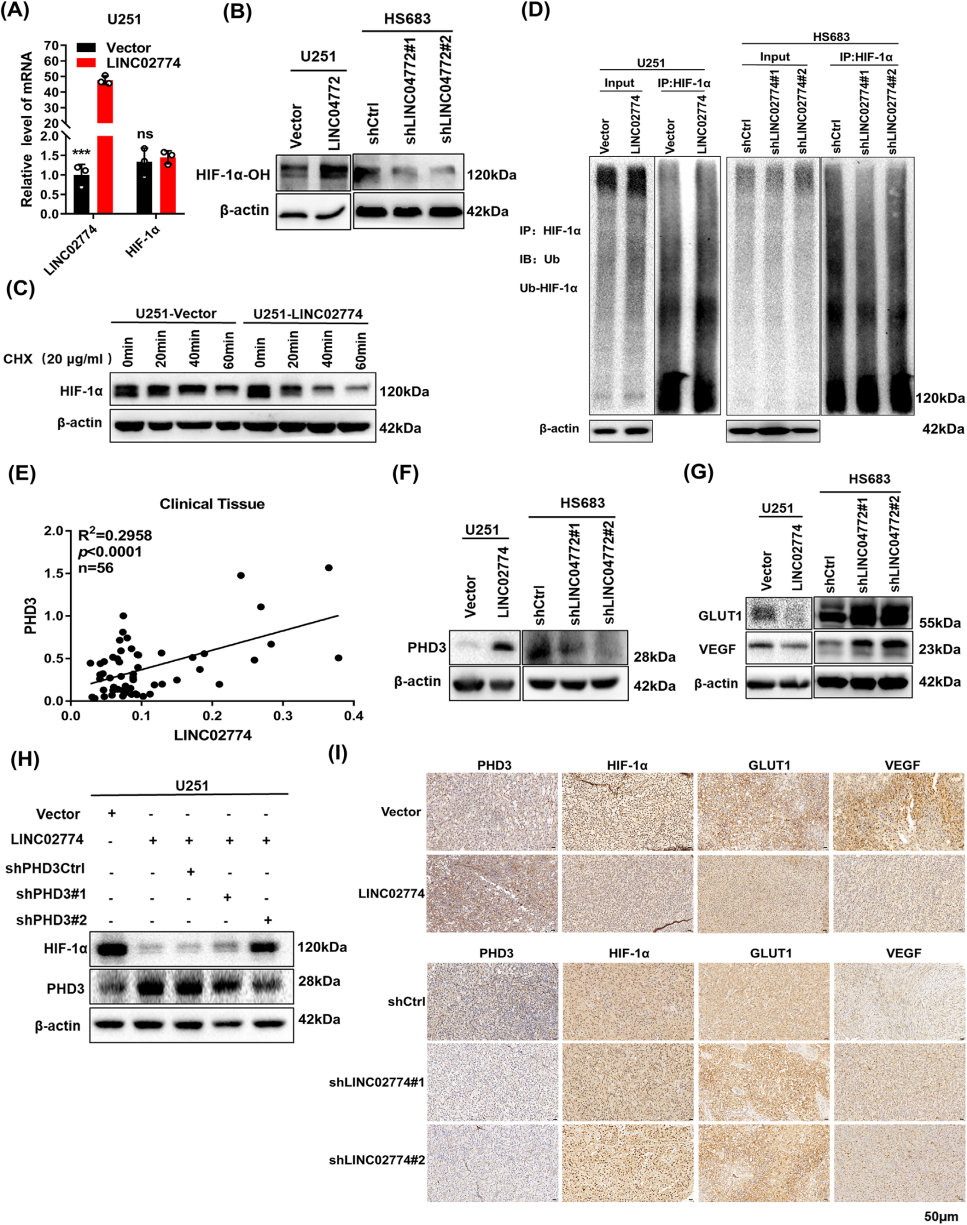
RIEMR‐associated LINC02774 promotes the degradation of HIF‐1α by upregulating PHD3. (A) Evaluate the transcription level of HIF‐1α in U251 cells stably overexpressing LINC02774 compared to vector. (B) LINC02774 promoted the hydroxylation of HIF‐1α in U251 cells overexpressing LINC02774 cultured in hypoxia. The hydroxylation of HIF‐1α was downregulated in HS683 with LINC02774 depletion and was cultured under hypoxia for 24 h. (C) LINC02774 overexpression accelerated HIF‐1α protein degradation. Cycloheximide (CHX, 20 μg/mL) was used to treat the LINC02774‐overexpressing U251 cells. (D) The ubiquitination of HIF‐1αwas detected by using an anti‐Ub antibody. (E) Correlations between LINC02774 and PHD3 in clinical glioma tissues (*n* = 56). (F) Western blot detected the expression of PHD3 in U251 and HS683 cells after overexpressing or depleting LINC02774. (G) Detect the expression of VEGF and GLUT1 in U251 cells overexpressing LINC02774 or LINC02774‐knockdown in HS683 cells. (H) Western blot to evaluate the expression of HIF‐1α in LINC02774‐overexpressing U251 cells transiently transfected with PHD3‐knockdown shRNA plasmid and cell lysates were captured at 72 h. (I) IHC analysis was performed to identify the expression levels of PHD3, HIF‐1α, VEGF, and GLUT1 in xenograft tumors, which are genes regulated by LINC02774 in cell lines. IHC: Immunohistochemistry.

To investigate the underlying mechanism that the LINC02774 downregulates the hypoxia and glycolysis pathway in glioma. Interestingly, the LINC02774 specifically downregulated the protein of HIF‐1α without affecting its transcription, as confirmed by qRT‐PCR analysis (Figure 5A), suggesting that LINC02774 may modulate the stability of HIF‐1α rather than directly affecting its transcriptional activity. To investigate potential mechanisms by which LINC02774 affects the stability of HIF‐1α, the western blot experiment was performed to verify whether the LINC02774 increased the hydroxylation level of HIF‐1α to promote degradation. Indeed, the overexpression of LINC02774 increased the hydroxylation of HIF‐1α in U251 (Figure 5B). Moreover, the depletion of LINC02774 inhibited the LINC02774‐induced hydroxylation of HIF‐1α in HS683 cells (Figure 5B). To further address whether LINC02774 can downregulate the protein of HIF‐1α by accelerating its degradation, we detected the HIF‐1α protein degradation rate using a cycloheximide (CHX) chase assay in U251 cells with overexpressed LINC02774. As anticipated, the HIF‐1α protein half‐life was decreased after overexpressing LINC02774 (Figure 5C). Furthermore, overexpression of LINC02774 strongly promoted HIF‐1α ubiquitination in the U251 cell line, and knockdown of LINC02774 dampened the ubiquitination levels of HIF‐1α (Figure 5D). These findings demonstrated that LINC02774 could reduce the stability of HIF‐1α by promoting hydroxylation and ubiquitination to degrade.

It is well established that the dioxygenase prolyl hydroxylases (PHDs) can catalyze the hydroxylation of HIF‐1α,^30^ PHDs includes three paralogs, among which PHD2 appears to be the main HIF‐1α hydroxylase.^19^, ^31^ We investigated whether LINC02774 regulates the expression of PHD2. However, the expression of PHD2 did not affect by LINC02774 in glioma cell lines (Figure S5A and B). It has been previously reported that the lncRNA‐HISLA stabilizes HIF‐1α by interfering PHD2 interaction with HIF‐1α.^13^ This raised the question of whether LINC02774 enhances HIF‐1α hydroxylation through enhancing the interaction between HIF‐1α and PHD2. However, the co‐immunoprecipitation (Co‐IP) experiment showed no change in the interaction between HIF‐1α and PHD2 in U251 cells overexpressing LINC02774 (Figure S5C and D). Thus, these findings suggest that PHD2 is not related to the signaling pathway, which LINC02774 downregulates the expression of HIF‐1α and promotes its hydroxylation and ubiquitination.

PHD3 is also known to regulate the hydroxylase and degradation of HIF‐1α.^32^ Next, we investigated whether LINC02774 regulates PHD3 expression. Analysis of LINC02774 and PHD3 mRNA in 695 glioma samples from the TCGA revealed a positive correlation between their expression levels (Figure S5E). A similarly relationship was observed in 56 clinical glioma tissues (Figure 5E). Notably, overexpression of LINC02774 enhanced the protein level of PHD3 (Figure 5F), while depletion of LINC02774 reduced PHD3 expression in HS683 cells (Figure 5F). These findings support the notion that LINC02774 regulates PHD3 expression, thereby influencing the hydroxylation and ubiquitination of HIF‐1α.

Then, we explored whether LINC02774 could suppress the expression of VEGF and GLUT1, the target genes of HIF‐1α.^33^ The LINC02774 overexpression inhibited the expression of VEGF and GLUT1 were been validated (Figure 5G). Conversely, the depletion of LINC02774 in HS683 cells resulted in increased the expression of VEGF and GLUT1 (Figure 5G). Importantly, we performed a rescue experiment and observed that LINC02774 downregulated the expression of HIF‐1α. However, when PHD3 was depleted in U251 with stable LINC02774 overexpression, the expression of HIF‐1α was no longer downregulated (Figure 5H), suggesting that the expression of HIF‐1α regulated by LINC02774 is dependent on PHD3.

And overexpression of LINC02774 resulted in downregulation of VEGF and GLUT1, while depletion of LINC02774 led to upregulation of these genes in xenograft tumors, as detected by Immunohistochemistry (IHC) analysis (Figure 5I). These findings provide further evidence that LINC02774 downregulates the hypoxia pathway both in vivo and in vitro.

### 2.6 RP58 involves in RIEMR‐associated LINC02774 regulates the PHD3 and suppresses the hypoxia signaling activity

To further explore the detailed mechanism by which LINC02774 upregulates the protein levels of PHD3, we investigated whether LINC02774 may have similar functions as lincRNA‐p21 in predominantly upregulate its neighboring gene,^34^ and LINC02774 through activating the expression of RP58 to upregulate the protein levels of PHD3. Analysis of the UCSC database confirmed that LINC02774 is located close to RP58 (Figure S6A). RP58 was also function as a brain tumor suppressor, but the mechanism is still unclear.^25^, ^35^ Importantly, RP58 exhibited high specificity in brain tissue（from the UCSC database） and decreased expression in glioma with increasing malignancy (Figure S6B and C), further supporting its role as a tumor suppressor in glioma. We then conducted experiments to investigate whether LINC02774 regulates the expression of RP58. However, overexpression of LINC02774 did not significantly change the levels of RP58 mRNA or protein (Figure S6E and F). Additionally, we examined whether RP58 regulates the expression of LINC02774 by overexpressing RP58 in U251 cells, but no effect on the expression of LINC02774 was observed (Figure S6G and H). These results suggest that LINC02774 and RP58 do not regulate each other's expression.

**FIGURE 6:**
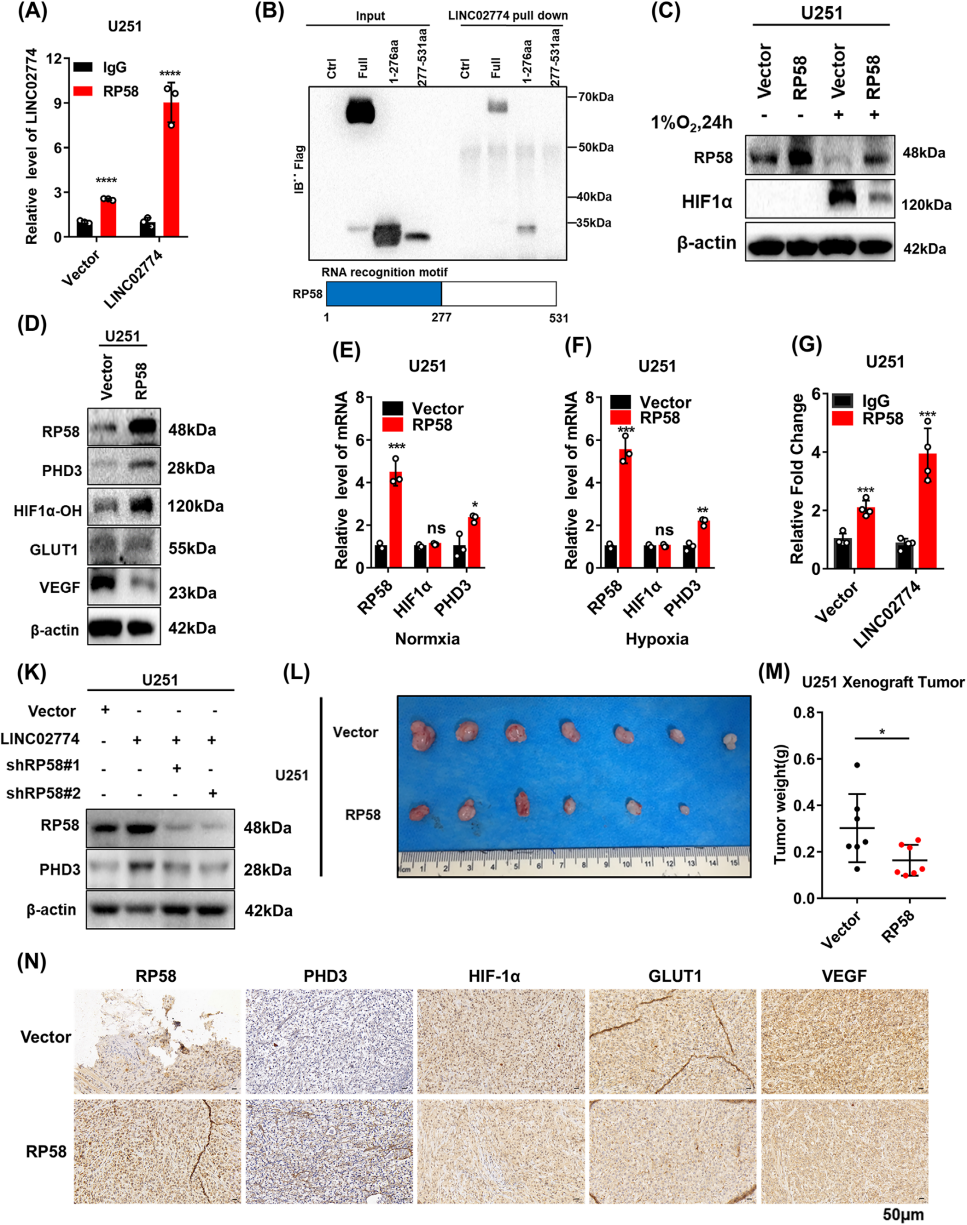
RP58 in RIEMR‐associated LINC02774 regulates the PHD3 and suppresses the hypoxia signaling activity. (A) RIP assay was used to detect the level of LINC02774 binding to RP58 under endogenous or exogenous LINC02774 derived from U251 transfected with LINC02774 or vector. (B) RNA pull‐down analysis of FLAG‐tagged RP58 (Full‐length, 1−276aa vs 277−531aa) pull‐down. (C) Western blot to detect the expression level of HIF‐1α in U251 cells after overexpressing RP58. (D) The level of PHD3, HIF‐1α‐OH, GLUT1 and VEGF in U251 cells overexpressing RP58. (E, F) The transcription level of PHD3 and HIF‐1α following the overexpression of RP58 were identified in the U251 cell line as well as culture in 21% (normoxia)(E) or 1%O2 (hypoxia)(F) for 24 h. (G) ChIP analysis of RP58 binding to PHD3 promoters in U251cell transfected with LINC02774 or vector. (H) The expression of PHD3 in LINC02774‐overexpressing U251 cells transiently transfected with RP58‐knockdown shRNA plasmid, and cell lysates were harvested at 72 h. (I, J) The xenograft tumors were shown in nude mice. Tumor images (I) and weights (J) were recorded (*n* = 7 for each group). (K) The expression levels of RP58, PHD3, HIF‐1α, VEGF, and GLUT1 identified by IHC in xenograft tumors.

Intriguingly, RNA immunoprecipitation (RIP) assays demonstrated an interaction between LINC02774 and RP58 (Figure 6A). To identify the specific region of RP58 that interacts with LINC02774, RNA pull‐down assays were carried out utilizing truncated variants of RP58. The results revealed that the RNA recognition motif (RRM) domain of RP58 (amino acids 1−276) exhibited a higher relative enrichment with LINC02774 (Figure 6B), indicating that the 1−276 amino acid region of RP58 forms a stable complex with LINC02774.

To explore the potential mechanism of the LINC02774‐RP58 interaction in regulating PHD3 expression and subsequently modulating the hypoxia and glycolysis signaling pathway, the mRNA expression of RP58 and PHD3 were analyzed in 695 glioma samples from the TCGA database. The analysis demonstrated the mRNA expression levels of PHD3 were positively correlated with RP58 in TCGA (Figure S6D). We further examined whether RP58 regulates the expression of HIF‐1α and PHD3 in U251 glioma cells by overexpressing RP58 using lentivirus. Remarkably, RP58 overexpressed significantly downregulated the protein of HIF‐1α (Figure 6C). Moreover, RP58 upregulated the PHD3 expression and facilitated HIF‐1α hydroxylation, leading to the suppression of VEGF and GLUT1 expression (Figure 6D). These results suggest that RP58 affect the hypoxia and glycolysis pathway, consistent with the function of LINC02774. Since RP58 is a transcription factor, we explored whether RP58 could regulate the mRNA levels of PHD3. We found that RP58 increased the mRNA levels of PHD3 but did not affect HIF‐1α mRNA levels (Figure 6E and F). Chromatin IP (ChIP) assays were conducted to examine the binding of RP58 at PHD3 promoters in U251 cells after overexpression of LINC02774, and the results demonstrated increased enrichment of RP58 at PHD3 promoters (Figure 6G). These findings revealed that LINC02774 promotes the RP58 binding to the promoter of PHD3, result in the upregulation of PHD3 protein levels and subsequent catalysis of HIF‐1α hydroxylation and degradation. To further elucidate the relationship between LINC02774, RP58, and PHD3, a rescue experiment was performed. The results revealed that LINC02774 upregulated the protein of PHD3, while depletion of RP58 in the stably overexpressing LINC02774 cells resulted in downregulation of PHD3 expression (Figure 6H), suggesting that LINC02774 regulates the expression of PHD3 in a manner dependent on RP58.

We carried out an experiment in which nude mice were injected with U251‐RP58 and U251‐Vector cells. We observed that overexpression of RP58 significantly inhibited tumor size and weight (Figure 6I and J). IHC analysis of xenograft tumors revealed that RP58 upregulated PHD3 expression and downregulated the expression of HIF‐1α, VEGF, and GLUT1 (Figure 6K). These results indicate that RP58 inhibits tumor growth in vivo and downregulates the hypoxia pathway both in vivo and in vitro.

### 2.7 RIEMR‐associated LINC02774 is a biomarker for prognosis and progression in glioma patients

**FIGURE 7:**
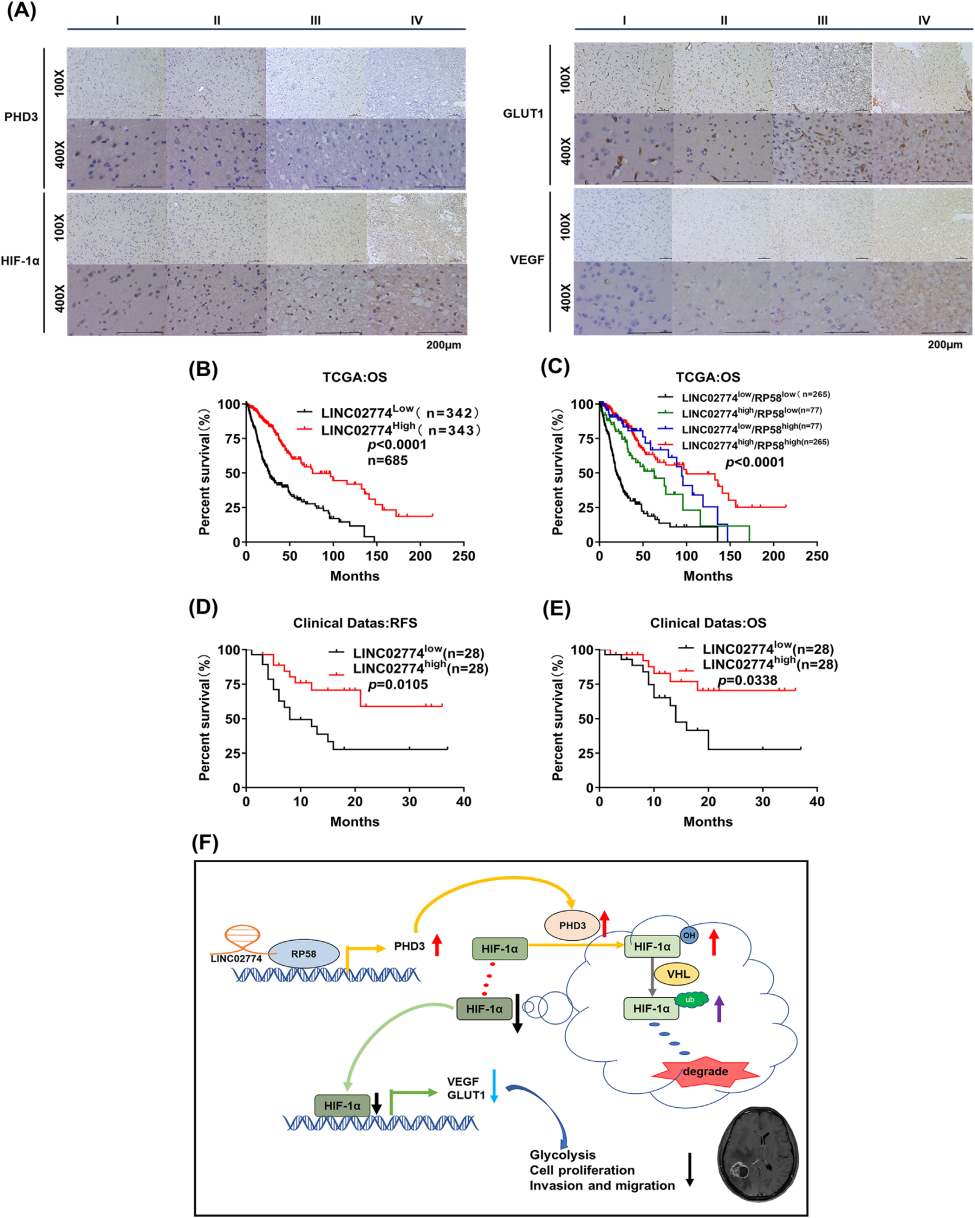
RIEMR‐associated LINC02774 is a biomarker for prognosis and progression in glioma patients. (A) Immunohistochemistry staining detection of PHD3, HIF‐1α, GLUT1 and VEGF proteins in glioma along with different WHO grades (I, II, III and IV). (B) Kaplan–Meier analysis was conducted to evaluate the OS curves of glioma patients based on the expression levels of LINC02774. The TCGA database was utilized, and the specimens were divided into two groups: low LINC02774 expression (*n* = 342) and high LINC02774 expression (*n* = 343). (C) Kaplan–Meier analysis was used to assess the OS in glioma patients based on the expression levels of both LINC02774 and RP58. (D, E) Clinical follow‐up was conducted on 56 glioma patients, the OS(D) and RFS (E) associated with LINC02774. (F) A schematic model illustrating the major molecular mechanisms of the “LINC02774/RP58/PHD3/HIF‐1α” axis in glioma is shown. OS: overall survival, RFS: recurrence‐free survival.

To confirm the clinical significance of LINC02774 and examine whether the downstream targets of LINC02774 exhibit consistent changes in clinical glioma specimens, we analyzed the expression levels of LINC02774, PHD3, RP58, HIF‐1α, VEGF, and GLUT1 in different grades of glioma tissues. Consistent with the in vitro findings, we observed that the highest expression of LINC02774 in grade I glioma (Figure 1B, D, and E). Additionally, we found that the density of PHD3 and RP58 decreased with increasing tumor grades, while the expression of HIF‐1α, VEGF, and GLUT1 showed an opposite trend (Figures 7A and S7A). These results, obtained through IHC analysis, indicate that the downstream targets of LINC02774 exhibit consistent changes in glioma patients.

The clinical data from the TCGA database was used to assess the clinical prognostic relevance of LINC02774 and RP58 in glioma patients. We found that the higher expression of LINC02774 was related to the longer overall survival (OS) in glioma patients (Figures 7B and S7B and C). Similar results were observed for RP58 (Figure S7D). Additionally, based on the combination expression of LINC02774 and RP58, we demonstrated that glioma patients with high expression of both LINC02774 and RP58 tended to have longer survival, while patients with differential expression of the two genes had intermediate prognostic outcomes. The worst prognosis was identified in patients with low expression of both variants (Figure 7C). Furthermore, we examined the expression levels of LINC02774 and performed follow‐up in 56 glioma patients. The results shown that decreased the expression of LINC02774 in glioma was related to poor OS in patients (Figure 7D), consistent with the TCGA data. Additionally, the higher expression of LINC02774 was associated with the better recurrent‐free survival (RFS) (Figure 7E). These findings suggest that high expression of LINC02774 is associated with a favorable prognosis.

Altogether, our analysis of human glioma clinical data demonstrated that LINC02774 is downregulated in glioma and can serve as a prognostic and progression indicator of clinical outcomes in glioma patients.

## 3 DISCUSSION

We found that RIEMR‐associated LINC02774 interacts with RP58, leading to the upregulation of PHD3 expression, which, in turn, facilitates the hydroxylation and ubiquitination of HIF‐1α in this study. Consequently, the degradation of HIF‐1α is promoted, resulting in the downregulation of GLUT1 and VEGF expression. Ultimately, this leads to the inhibition of the glycolysis pathway and the progression of glioma (Figure 7F).

It has been discovered that lncRNAs perform a variety of roles in cancer. With the advancements in RNA‐sequencing technologies, novel cancer‐associated lncRNAs can be identified.^7^ The function of lncRNAs can vary depending on their subcellular localization and expression levels.^36^ Coincidentally, the LINC02774 is located in the nucleus, which may perform a variety of roles in regulating the transcription of genes in cancer, such as transcriptional regulation *in cis* and *in trans*.^6^, ^27^, ^37^ Additionally, lncRNAs can function *in cis* to upregulate the expression of neighboring genes.^7^ These findings prompted us to explored the potential role of LINC02774 in glioma. Our study provides compelling evidence supporting the tumor suppressor role of LINC02774 in glioma. We have also identified its interaction with the neighboring gene RP58, and together they coordinate the regulation of downstream pathways.

The involvement of lncRNAs in hypoxia‐associated malignant progression has been extensively studied, leading to the identification of several hypoxia‐responsive lncRNAs.^38^, ^39^, ^40^ Interestingly, LINC02774 does not exhibit a response to hypoxia. However, it interacts with RP58 and subsequently upregulates PHD3, which in turn catalyzes the hydroxylation and ubiquitination of HIF‐1α, ultimately destabilizing HIF‐1α. HIF‐1α is a key factor in the hypoxia pathway, and its exaggerated expression can lead to the Warburg effect.^41^ Thus, there is a close interplay between the pathway of hypoxia and glycolysis. In our study, we found that LINC02774 inhibits both the hypoxia pathway and glycolysis, and interestingly, its expression is not influenced by the hypoxic condition.

The accumulation of HIF‐1α leads to the transcription of genes involved in hypoxia‐related processes, contributing to tumorigenesis and angiogenesis. Activation of the HIF‐1α pathway is associated with aggressive tumor characteristics and unfavorable clinical outcomes in various cancer types.^42^ Here, we also demonstrated that the expression of LINC02774 correlated with prognosis, clinicopathologic and the manifestations of malignant degree on MRI. Thus, LINC02774 could be considered a biomarker for determining the prognosis and progression in glioma patients.

However, had certain drawbacks in this research. First, samples taken from 56 individuals were used to examine clinical data, which resulting in biased statistical. Second, although the expression of LINC02774 was accessed by the qRT‐PCR, additional tests are required to confirm its expression in glioma tissue. Third, the relationship among LINC02774, PHD3, and HIF‐1α should be demonstrated in glioma tissue.

Our research provides novel insights into the function of RIEMR‐associated LINC02774 as a tumor suppressor in glioma. We have demonstrated its inhibitory effects on the hypoxia and glycolysis pathway and its ability to suppress tumor cell proliferation. These findings highlight the significance of RIEMR‐associated LINC02774 in tumor progression and suggest its potential as a therapeutic target for glioma.

## 4 MATERIALS AND METHODS

### 4.1 Human clinical specimen, IHC, and H&E

In this research, we collected two sets of human glioma specimens for analysis. The first set comprised 56 fresh frozen glioma tissues (grades II, III, or IV), including 13 pairs of glioma specimens and corresponding adjacent normal cerebral tissues. These samples were used to assess the expression of LINC02774 and PHD3 using qRT‐qPCR. The second set consisted of 20 paraffin‐embedded glioma tissue specimens (grade I, II, III, or IV), which were utilized to validate the expression of LINC02774, RP58, PHD3, HIF‐1α, GLUT1, and VEGF. The protocol for analyzing the IHC data was as previously described,^43^ sections were cut with 4 μm thickness from each paraffin tissue, tissue sections underwent deparaffinization and hydration, 3% hydrogen peroxide (H_2_O_2_) treatment, and heating in sodium citrate for antigen retrieval. Then, antibodies used were as follows: HIF‐1α (Novus, # NB100‐105, 1:40), PHD3 (Novus, #NB100‐139, 1:500), GLUT1 (Proteintech, # 66290‐1‐Ig, 1:400), RP58 (Proteintech, #12714‐1‐AP, 1:4000), and VEGF (Proteintech, # 19003‐1‐AP, 1:400), 4°C overnight, then incubated with the secondary antibody. The result of the stained slides was independently examined by two pathologists (Xiao D and Li L). All glioma samples from the Xiangya Hospital of Central South University (Changsha, China) and underwent histopathological examination.

Specimens were fixed in formalin for 24 h. They were then transferred to 70% ethanol before being stained with H&E.

### 4.2 Bioinformatics analysis

Expression data of glioma was obtained from the TCGA database. Differentially expressed lncRNAs were determined based on a cutoff fold change of ≥1.5 and a *p* value < 0.001. Additionally, the GEPIA online database (http://gepia.cancer‐pku.cn/) was utilized to investigate glioma‐associated lncRNAs and perform Kaplan–Meier survival analyses. Gene expression was visualized using the UCSC genome browser database (https://genome.ucsc.edu/). CpG islands of LINC02774 were obtained from the UCSC database, while methylation data was acquired from the TCGA. The *R* software was employed to analyze the TCGA gene expression profile data and methylation levels.

### 4.3 Measurement of the relative index of enhanced magnetic resonance (RIEMR)

Qualitative and semiquantitative imaging analysis methods were employed as previously described.^44^, ^45^, ^46^, ^47^ A region of interest (ROI) was selected, and the MR signal intensity (SI) values were measured in the contrast‐enhanced T1‐weighted images (SI _tumor_). In the same MRI slice, an ROI was drawn in the brain tissue symmetrical to the tumor, and the SI was measured (SI _normal brain_). Three paired SI measurements were obtained from different slices of the contrast‐enhanced T1‐weighted images. RIEMR was then calculated using the following formula: RIEMR = SI _tumor_ / SI _normal brain_.

### 4.4 Cell cultures, chemicals, plasmids, and shRNAs

ATCC provided the HS683, U251, and U87‐MG cell lines, which were cultured in DMEM medium (Gibco, Switzerland) supplemented with 10% fetal bovine serum. To induce hypoxia, the Hypoxic Workstation (Don Whitley, UK) was used to culture the glioma cells for 24 h. RNA and protein extracted from the hypoxia‐treated cells was performed within the workstation, while the medium collected from the cells was used for other experiments.

Lentiviral shRNA clones targeting human LINC02774, RP58, and a nonspecific control (GV248) were obtained from GeneChem (Shanghai, China). LINC02774 and RP58 overexpressing plasmids were constructed by cloning LINC02774 or RP58 cDNA into the pLVX‐EF1α‐IRES‐Puro vector (cat#631988, Clontech). Lentiviral particles were produced using 293T cells. Lentiviral particles were used to infect HS683, U251, and U87‐MG cell lines and subsequently selected with puromycin at 2 μg/mL. Table S1 displayed the sequences of the specific shRNAs.

### 4.5 RNA isolation and qRT‐PCR

RNAiso Plus (Takara) and PrimeScript™ RT reagent kit with gDNA Eraser (Takara) were used to extract the gRNA and reverse transcribed into cDNA, following the manufacturer's protocol. The expression level of β‐actin was used to normalize the relative gene expression. In Table S2, the corresponding primers used in this experiment are listed.

### 4.6 RNA‐sequencing analysis

Total RNAs were extracted from U251 glioma cells that stably overexpressed LINC02774 or vector. The RNA‐sequencing process, including the differentially expressed genes and the analysis of KEGG and GO pathways, was conducted by BGI Tech (Shenzhen, China) (https://report.bgi.com/ps/mrna/index.html). GSEA was also performed to analyze the pathways associated with the differentially expressed gene sets between the two groups.

### 4.7 Western blots and Co‐IP assay

IP lysis buffer with a protease inhibitor cocktail was used to harvest the protein from the cells. 40 μg of total protein was transferred onto a PVDF membrane. The membrane was subjected to immunoblotting by incubating with specific primary antibodies overnight at 4°C. Antibody signals were detected by ChemiDox XRS+.

For immunoprecipitation experiments, cells were harvested, lysed, quantified, and precleared with 20 μL of Dynabeads™ Protein G (ThermoFisher Scientific, #10004D, USA). After centrifugation, the supernatant was collected and incubated with antibody at 4°C overnight. Dynabeads™ Protein G was added to the protein‐antibody complex for additional incubation. Subsequently, the beads were collected and boiled. Western blot analysis was performed on the samples, with 20 μg of total protein used as the input control.

The antibodies for Western Blot: HIF‐1α (CST, #36169S, 1:1000), Hydroxylation HIF‐1α (CST, #3434T, 1:1000), PHD3 (Novus, #NB100‐139, 1:1000), GLUT1 (CST, #12939S, 1:1000), RP58 (Proteintech, #12714‐1‐AP, 1:1000), PHD2 (CST, #4835S, 1:1000), VEGF (Abclonal, #A12303, 1:500), N‐cadherin (CST, #13116, 1:1000), HK2 (Proteintech, #22029‐1‐AP, 1:2000), Ubiquitin (CST, #3936S, 1:1000), PGK1 (Proteintech, #17811‐1‐AP, 1:1000), β‐actin (Sigma, #A5441, 1:10000), ABCG2 (Sigma‐Aldrich, MAB4146, 1:1000), and anti‐Flag (CST, #14793, 1:1000).

### 4.8 Cell proliferation assays, Transwell assays, and clonogenic assays

The detailed protocols of these experiments were as previously reported.^27^, ^28^ Briefly, 500 cells were seeded plate and cell viability was assessed by MTS kit.

Cells were seeded into the upper chamber of Transwell inserts and placed in 24‐well plates, and cells that had migrated to the bottom of the Transwell insert were counted under a microscope.

500 cells were seeded into individual wells of 6‐well plates and cultured continuously for 2 weeks to allow colony formation, counted the colony by using the ImageJ software.

### 4.9 RIP assays

Glioma cells were lysed in RIP buffer, and the lysates were divided into three fractions. Antibodies against normal rabbit IgG (CST, #2729) and RP58 (Proteintech, #12714‐1‐AP) were added to the appropriate fractions and overnight at 4°C. On the following day, 40 μL of Dynabeads™ Protein G (ThermoFisher Scientific, #10004D, USA) were washed and added to the protein‐RNA complexes for further incubation and rotation. The beads were collected and washed, followed by one wash with PBS. Next, 1 mL of RNAiso Plus (Takara) was added to resuspend the beads and isolate the RNA for qRT‐PCR analysis.

### 4.10 ChIP assays

A total of 1.0 × 10^7^ glioma cells were fixed in formaldehyde (Sigma, #SZBF1830V). Glycine was added to stop the fixation. The cells were then subjected to sonication using a Qsonica sonicator, with 6 min of alternating cycles of 20 s on and 20 s off, and the supernatant was collected. Each immunoprecipitation reaction utilized 300 μg. Then, 2.0 μg of anti‐RP58 antibody (Proteintech, #12714‐1‐AP) and 2.0 μg of normal rabbit IgG were added and incubated overnight. The preblocked Dynabeads™ Protein G (ThermoFisher Scientific, #10004D, USA) was used to capture the antibody‐protein complex. ChIP DNA was subsequently analyzed by qRT‐PCR: Forward: 5′‐TTCAGTCTGCTTTCCAG‐3′ and Reverse: 5′‐AAGCCACCACTGCCGCGACT‐3′.

### 4.11 RNA pull‐down assays

The LINC02774 was transcribed by using the TranscriptAid T7 High Yield Transcription Kit (ThermoFisher Scientific, # K0441) in vitro. The full‐length LINC02774 was biotinylated using the Pierce™ RNA 3′ End Desthiobiotinylation Kit (ThermoFisher Scientific, #20163). The cellular protein extracts from 293 T cells. Magnetic RNA‐Protein Pull‐Down Kit (ThermoFisher Scientific, #20164) was used to the RNA pull‐down assay.

### 4.12 Glucose uptake and lactate production

The glucose and lactate in the medium were measured by Automatic Biochemical Analyzer (7170A; HITACHI).

### 4.13 Nude mice assay

Four‐week‐old female BALB/c athymic mice were purchased from the Hunan SJA Laboratory Animal Co. Ltd. The xenograft tumor formation assay was performed as previously described.^2^, ^27^, ^48^ Briefly, 5×10^5^ cells in 100 μL PBS were subcutaneously injected into the mice. The length and width of the tumor were measured every 3 days, and the tumor volumes were calculated.

The procedure of the orthotopic in vivo model was performed as previously described.^49^ Briefly, glioma cells (1×10^5^) resuspended in 3 μL PBS were injected into the brain. The mice's intracranial tumor size was assessed by MRI, and they were euthanized after 28 days.

### 4.14 Statistical analyses

All experiments, except those involving mice, were repeated at least three times. The results are presented as the mean ± SEM. Statistical differences between the two groups were determined using Student's *t*‐test (two‐tailed). Prism 7.0 GraphPad software was used for all statistical analyses. A *p* value less than 0.05 was considered statistically significant. Where *ns* denotes nonsignificant (*p* > 0.05), *p < 0.05, **p < 0.01, ***p < 0.001, and ****p < 0.0001.

## AUTHOR CONTRIBUTIONS

YT and JH designed the study and wrote the paper. JH and YC performed cell experiments and analyzed data. YC, YL, and LO performed in vitro biochemical experiments. JX and SL performed the glucose and lactate measurements. JH, CM, and MT performed in vivo experiments. ZY performed bioinformatics analysis. LL and DX acquired and analyzed clinical specimens. The final manuscript was read and approved by all authors.

## CONFLICT OF INTEREST STATEMENT

The authors have declared that no conflict of interest exists.

## ETHICS STATEMENT

All procedures for the animal study were approved by the Institutional Animal Care and Use Committee of the Central South University of Xiangya School of Medicine and conformed to the legal mandates and federal guidelines for the care and maintenance of laboratory animals (2020sydw0170). All patient glioma samples were collected from the Xiangya Hospital of Central South University (Changsha, China). All study participants had written informed consent. This study was approved by the Research Ethics Committee of the Xiangya hospital (202004150).

## Supporting information

Supporting Information

## Data Availability

All the data generated or analyzed during this study are included in this published article and its supplementary files.
