# Supplementary material for: LINC02774 inhibits glycolysis in glioma to destabilize HIF‐1α dependent on transcription factor RP58
Source: MedComm (2020). 2023 Sep 11;4(5):e364. doi: 10.1002/mco2.364 (PMC10494996; doi:10.1002/mco2.364)
Supplement: Supplementary file 1 — Supporting Information [file MCO2-4-e364-s001.docx]

**LINC02774 inhibits glycolysis in glioma to destabilize HIF-1α dependent on transcription factor RP58**

Yuanbing Chen^1,2,3^, Yating Liu^4,5^, Jianbing Xiong^6^, Lianlian Ouyang^4,5^, Miao Tang^23^, Chao Mao^4,5^，Liling Li^7^, Desheng Xiao^7^, Shuang Liu^3,8^, Zhen Yang^9^，Jun Huang^2,3,*^，Yongguang Tao^4,5,*^

^1^ Department of Neurosurgery, The Third Xiangya Hospital of Central South University, Changsha, China, 410006

^2^ Department of Neurosurgery, Xiangya Hospital, Central South University, Changsha, China，410008.

^3^ National Clinical Research Center for Geriatric Disorders, Xiangya Hospital, Central South University, Changsha, Hunan, China, 410008.

^4^Key Laboratory of Carcinogenesis and Cancer Invasion, Ministry of Education, Central South University, Hunan, China, 410008.

^5^NHC Key Laboratory of Carcinogenesis (Central South University), Cancer Research Institute, Central South University, Changsha, Hunan, China,410008.

^6^Department of Emergency, Xiangya Hospital, Central South University, Changsha, Hunan, China，410008.

^7^Department of Pathology, Xiangya Hospital, Central South University, Changsha, Hunan, China, 410008.

^8^Department of Oncology, Xiangya Hospital, Central South University, Changsha, China，410008.

^9^Shanghai Key Laboratory of Medical Epigenetics, Fudan University, Shanghai 200032, China.

* Corresponding author: Jun Huang: [xyyyhj@csu.edu.cn](mailto:xyyyhj@csu.edu.cn); Yongguang Tao: [taoyong@csu.edu.cn](mailto:taoyong@csu.edu.cn).

Yongguang Tao, ORCID ID: 0000-0003-2354-5321

Jun Huang, ORCID ID: 0000-0002-1942-6787

**Supplemental Figure Legends**

**Figure S1**

**Figure S1. LINC02774 is downregulated in human glioma tissues**

**A**) The volcano map showed the differentially expressed genes between GBM and normal brain tissues in the left, and the differentially expressed genes between GBM and LGG in the right. **B**) The different expression level of LINC02774 between GBM and LGG in TCGA database. **C**) Downregulated the LINC02774 expression in glioma was analyzing by GEPIA. **D**) The LINC02774 is highly specific expressed in normal brain tissue showed in UCSC Genome Browser. **E**) The LINC02774 expression levels in different glioma cell lines (U251, U87-MG, HS683). **F**) The expression of LINC02774 in cytosolic and nucleus fractions derived from U251 and HS683 glioma cell lines. **G**) Through analyzing the TCGA database that implied the LINC02774 expression was negatively correlated with the methylation level of CpG. **H**) Receiver-operating characteristic (ROC) curves displaying the sensitivity and specificity of LINC02774 expression for predict relative index of enhanced magnetic resonance (RIEMA). Insets indicate AUC values, 95% confidence intervals, and statistics.

**Figure S2**

**Figure S2. Overexpression of RIEMR associated LINC02774 attenuates EMT and glioma formation**

**A**) Expression of EMT markers N-cadherin, Sail and β-catenin in U251 cell overexpressing LINC02774 compared to control cells. **B**) Photos of xenograft tumors on nude mice (n=10 each group). **C**) The image of orthotopic xenograft tumors detected by MRI (n=5 each group).

**Figure S3**

**Figure S3. Knockdown of RIEMR associated LINC02774 promotes tumor formation**

**A**) Photos of xenograft tumors on nude mice which after injection of U87-MG cells stably transfected with two distinct LINC02774 shRNA (shLINC02774#1 and shLINC02774#2) vectors and control cells (shCtrl). (n=9 each group). **B**) The image of orthotopic xenograft tumors in cerebral with the U87-MG cells after the knockdown of LINC02774 and control cells and detected by MRI (n=5 each group).

**Figure S4**

**Figure S4. RIEMR associated LINC02774 alteration in glioma and RNA sequence data.**

**A-B**) GO functional classification(A) and enrichment(B) of differentially expressed genes for each pairwise comparison. **C-D**) KEGG classification(C) and enrichment(D) of differentially expressed genes for each pairwise comparison. **E-F**) RT-qPCR confirmed the sequencing results of mRNA.

**Figure S5**

**Figure S5. PHD2 did not involve in RIEMR associated LINC02774 downregulates the protein levels of HIF-1α**

**A-B**) Western blot assay was used to determine the expression level of PHD2 in U251 (A) and HS683 (B) cells after overexpression or depletion of LINC02774. **C-D**) Co-Immunoprecipitation (Co-IP) assay was performed to explore the LINC02774 effect on the interaction between PHD2 with HIF-1α in the U251 cell line. **E**) Correlations between LINC02774 and PHD3 in glioma tissues were analyzed based on TCGA gene expression profile data. **F**) Western blot assay was performed to explore the expression level of PGK1 and HK2 affected by LINC02774 in U251 cell line.

**Figure S6**

**Figure S6. The relationship between RP58 and RIEMR associated LINC02774**

**A**) Searched from the UCSC database and found that the LINC02774 neighbor with RP58. **B**) The expression of RP58 in different grades of WHO was analyzed from TCGA gene expression profile data (including grade II, n=311; III, n=208; IV, n=166). **C**) The RP58 is highly specific expressed in normal brain tissue showed in the UCSC Genome Browser. **D**) Correlations between RP58 and PHD3 in glioma tissues were analyzed from the TCGA gene expression profile data. **E-F**) The qRT-PCR (E) and Western blot (F) analysis were used to detect the expression of RP58 on the level of protein and mRNA affected by LINC02774 in U251 cell after overexpression of LINC02774. **G-H**) The qRT-PCR assay was performed to determine the expression of LINC02774 affected by RP58 in U251 cell stably overexpressing RP58 as well as culturing in 21%(normoxia) or 1%O_2_ (hypoxia) for 24 hours.

**Figure S7**

**Figure S7. RP58 is a biomarker for prognosis and progression in glioma patients**

**A**) Immunohistochemistry staining detection of RP58 proteins in glioma along with different Grade of WHO (Grade of I, II, III, IV). **B-C**) Kaplan–Meier analysis revealed overall survival (OS) curves of LGG(B) and GBM(C) patients with different expression of LINC02774. **D**) Kaplan–Meier analysis revealed overall survival (OS) curves of glioma patients with different expression of RP58 in TCGA database.

**Supplementary Table S1. The target sequence of shRNA**

The target sequence of shRNA

| shRNA | Sequence |
| --- | --- |
| LINC02774#1 | TTTGTACAGGAGCCATTTAAT |
| LINC02774#2 | GTTCTGTTAGCAAAGAATAAA |
| shRP58#1 | TCTCAAGAACTTTGGAAAT |
| shRP58#2  shPHD3#1  shPHD3#2 | TTCAAGTTGTTCGGACAAA  CACCTGCATCTACTATCTGAA  CTTGGTATTGAGCACGTATTT |

**Supplementary Table S2. Primers used for qRT-PCR**

qRT-PCR primers used for detection of the mRNA level

| Gene Name | Sequence |
| --- | --- |
| LINC02774 | F：ACATTGGAGACAGCATGGAG |
|  | R：TCTAAGGTGTTGGAGCTGCT |
| RP58 | F: CTGTCAAGTCCAGCCTTTCAGG |
|  | R: CACTCTCATCACAGGAAGCCTC |
| HIF-1α | F: TATGAGCCAGAAGAACTTTTAGGC |
|  | R: CACCTCTTTTGGCAAGCATCCTG |
| PHD3 | F: GAACAGGTTATGTTCGCCACGTG |
|  | R: CCCTCTGGAAATATCCGCAGGA |
